# Centromere histone H3- and phospholipase-mediated haploid induction in plants

**DOI:** 10.1186/s13007-019-0429-5

**Published:** 2019-04-26

**Authors:** Song Wang, Weiwei Jin, Kai Wang

**Affiliations:** 1 Key Laboratory of Genetics, Breeding and Multiple Utilization of Crops, Ministry of Education, Fujian Provincial Key Laboratory of Haixia Applied Plant Systems Biology, Fujian Agriculture and Forestry University, Fuzhou, 350002 Fujian China; 2 National Engineering Research Center of Sugarcane, Fujian Agriculture and Forestry University, Fuzhou, 350002 China; 3 Key Laboratory of Sugarcane Biology and Genetic Breeding, Ministry of Agriculture and Rural Affairs, Fujian Agriculture and Forestry University, Fuzhou, 350002 China; 4 College of Agriculture, China Agricultural University, No. 2, Yuan Ming Yuan West Road, Haidian District, Beijing, 100193 China

**Keywords:** Haploid induction, Centromere histone H3, Phospholipase gene, Centromere-size model

## Abstract

Simple and consistent production of haploid is always an appealing pursuit for both crop breeders and researchers. Although diverse strategies have been developed to produce haploids over the past decades, most of them are applicable in only a limited number of plant species. In 2010, Ravi and Chan reported that haploid *Arabidopsis thaliana* plants can be efficiently induced through the introduction of a single genetic alteration in centromere histone H3 (CENH3). Subsequent studies demonstrated that haploids can be efficiently induced either through genetic engineering of CENH3 N-terminal tail or histone fold domain or by replacing CENH3 with an ortholog. The mutation of a pollen-specific phospholipase gene, *MATRILINEAL* (*MTL*) has been revealed to trigger the haploid induction (HI) in maize, which present another promising HI approach by the editing of *MTL* in plant. Here, we review the progress of the CENH3-medialed HI and propose a revised centromere-size model by suggesting a competitive loading process between wild-type and mutant CENH3 during HI. This model can explain both the findings of HI failure when wild-type and mutant *CENH3* genes are coexpressed and the alien centromere loading of CENH3 in stable hybrids. In addition, we review the current understanding of *MTL*-mediated HI in plant. The conservation of *CENH3* and *MTL* in plants indicates wide potential application for HI. We discuss the utility and potential of these two methods in crops by comparing their mechanisms and applications to date in plants. This review will promote the study and application of both CENH3- and *MTL*-mediated haploid induction in plants.

**Electronic supplementary material:**

The online version of this article (10.1186/s13007-019-0429-5) contains supplementary material, which is available to authorized users.

## Background

The ability to generate haploid plants offers tremendous benefits in plant genetics and genomics, plant breeding, and embryology. Haploid plants can undergo genome doubling to produce genetically stable lines in a single generation; this method bypasses the traditional long-term inbreeding process and can be used in crops where self-pollination is not possible. In addition, haploids will simplify heritability studies due to having only one set of chromosomes, therefore recessive mutations could be easily identified. For these reasons, great efforts have been made over the past few decades to increase the efficiency of haploid production [1]. This review emphasizes on progress of two haploid induction (HI) methods: centromere histone H3 (CENH3)- and phospholipase gene (*MATRILINEAL*, *MTL*)-mediated. Due to the functional conservation of the *CENH3* and *MTL* genes, both methods promise extensive applicability in plants.

## Brief history of plant haploid induction

The first observation of a natural haploid in higher plants was in *Datura stramonium* by Bergner in 1922 [2]. Similar discoveries of haploids soon followed in other species, including *Nicotiana tabacum* [3] and *Triticum compactum* [4]. The potential importance of haploids in crop breeding and genetics was quickly recognized, resulting in the first boom in haploid production research. In the following decades, a wide range of haploid generation methods were found, including parthenogenesis (mainly via anther culture), pollen irradiation, seed selection with twin embryos, sparse pollination, alien cytoplasm and wide hybridization [5, 6]. Among these methods, anther culture and wide hybridization demonstrated the most promising.

Early attempts to create haploids from the male gametophyte resulted only in haploid callus tissues [7, 8]. Embryo-like haploids were first described in *Datura* by Guha and Maheshwari [9]. Although these androgenic haploids were not grown to maturity, this landmark research demonstrated the feasibility of haploid production by anther culture. Haploid plants were soon obtained from the cultured anthers of *N. sylvestris* and *N. tabacum* [10]. Thereafter, anther culture systems were successfully established in many plants. Moreover, a series of improved methods were also developed, including culture of microspores, ovaries and ovules [11]. To date, efficient protocols have been established to create haploids by anther culture or its derivatives in approximately 250 plants, including cereals, trees, and vegetables [12–14]. Thus, anther culture has become the preferred method of haploid plant production [13, 15].

Wide hybridization is another efficient way to generate haploids. In 1970, Kasha and Kao reported that the chromosomes of *Hordeum vulgare* were preferentially eliminated during embryo formation when crossing to bulb barley (*H. bulbosum*) [16]. Haploid plants were then obtained by embryo rescue, because the hybrid endosperm would often abort. Its genotype independence makes this method more promising than other haploid-producing methods in barley [17]. To date, over 60 barley cultivars have been produced around the world based on this strategy [13, 17]. Efficient HI was also found in wheat pollinated with maize, sorghum, barley, teosinte, and pearl millet [18–20]. However, this method currently has limited utility, functioning in only those few crops. Although reasons have been proposed for uniparental chromosome elimination, such as asynchronous cell cycle, formation of multipolar spindles, spatial separation of genomes during interphase, and nuclear extrusion [21–23], the actual cellular mechanism remains poorly understood. Based on detailed observation, Finch R.A. revealed that the centromeric constrictions of eliminated *Hordeum* chromosome were always either absent or much smaller than those of the retained chromosomes [24]. This finding provided the first association between centromere function and uniparental chromosome elimination in wide hybridization in *Hordeum*. Strikingly, a recent study indicated that the loss of CENH3 from *H. bulbosum* preceded uniparental chromosome elimination during the development of *H. vulgare* × *H. bulbosum* hybrid embryos [25]. This discovery of a key role for CENH3 in chromosome elimination suggested the possibility of producing haploids through CENH3 modification.

## CENH3-mediated haploid induction

CENH3 (called CENP-A in humans) is a centromere-specific histone H3 variant that replaces histone H3 in eukaryotic centromeric nucleosomes [26, 27]. CENH3 plays an essential role in the epigenetic formation of kinetochore [28, 29] and is sufficient to determine centromere identity, at least in *Drosophila* [30]. The key role of CENH3 in centromeres indicates that any error in transcription, translation, modification, or incorporation can affect the assembly of intact kinetochore and consequently may cause centromere dysfunction or inactivation [31].

In 2010, Ravi and Chan reported the breakthrough discovery that a CENH3 mutant can induce haploids when crossed to WT *Arabidopsis thaliana* [32]. This mutated CENH3 was created by replacing the endogenous CENH3 N-terminal tail with the green fluorescent protein (GFP)-fused N-terminal tail domain of H3.3 (tailswap). This modified *GFP*-*tailswap CENH3* gene can complement the null mutant *cenh3*-*1*, in which the native *CENH3* is knocked out. Strikingly, paternal haploid progeny can be obtained at a high rate (25–45% of viable offspring) when *GFP*-*tailswap* plants (as female) are crossed to WT plants. Haploid progeny can also be obtained (4–5%) when many *GFP*-*tailswap* anthers are used to pollinate WT plant (due to *GFP*-*tailswap* plants are almost completely male sterile). Meanwhile, a *GFP*-*CENH3* mutant (simply adding a GFP at the N-terminus of CENH3) was also shown to induce haploids (5%) when crossed to WT plants [32].

The distinctive feature of this CENH3-mediated HI is that the haploids are easily generated as seeds by crosses using the inducer (*GFP*-*tailswap* or *GFP*-*CENH3* mutants) as either female or male. This advantage facilitates wide variety of applications [33], including generating mapping populations [34], chromosomal substitution lines and parental lines for reverse breeding [35] and engineering clonal reproduction through seeds [36]. In addition, it suggests that plants with appropriate modifications of CENH3 can be used as haploid inducers to easily generate haploids. Subsequent studies have revealed that point mutations in the CENH3 α-N-helix or CATD (centromere-targeting domain) can also induce haploids [37, 38], as can the replacement of *Arabidopsis* CENH3 with those related species [39, 40].

## Strategies to modify CENH3 for HI

### N-terminal tail editing

The initial study of Chan and colleagues suggests that any modifications of CENH3 that can weaken centromere function can be used to produce haploids [32]. Thus, the key is to determine CENH3 regions related to centromere function. It is known that eukaryotic CENH3s have a highly variable N-terminal tail and a relatively conserved C-terminal region [27]. The N-terminal tail is extremely fast-evolving [41] and differs even between CENP-As from different species [42]. N-terminal targeting assays [43] and CENH3 and H3 domain swap experiments (where the regions of CENH3 are replaced by corresponding regions from H3) [32, 44–46] have revealed that the N-terminal tail is dispensable for its mitotic centromere function.

**Fig. 1:**
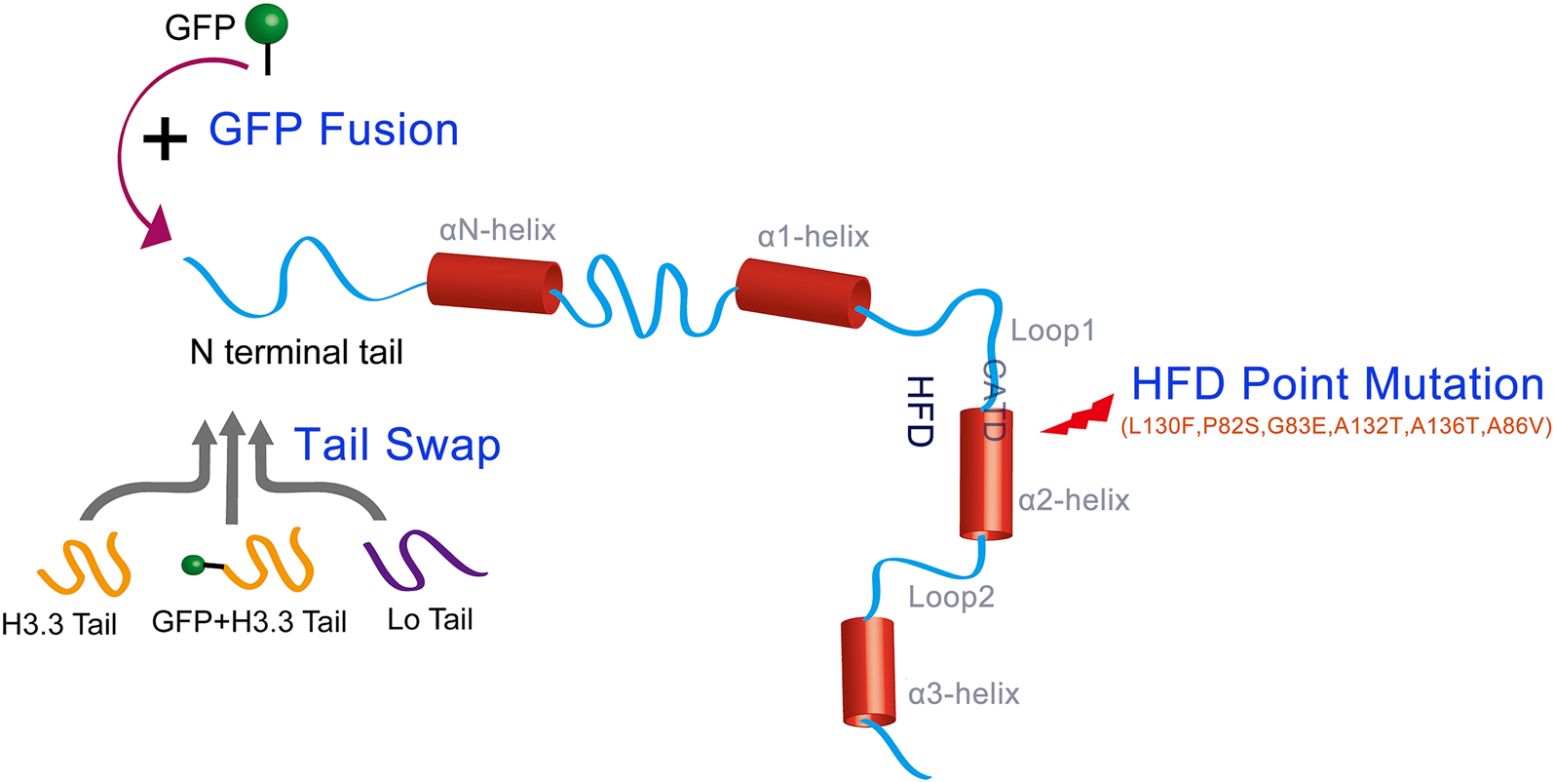
An illustration of the methods to modify CENH3 for HI. The modifications at N-terminal tail of CENH3 include direct conjugation of GFP tag (GFP Fusion) and replacement of the CENH3 tail with *L. oleraceum* tail as well as H3.3 tail with or without a GFP tag (Tail Swap). The modifications at C-terminus are mainly generated by the point mutation in the HFD region (HFD Point Mutation)

However, exchange of *A. thaliana* N-terminal tail between less related species (*maizetailswap*) will cause severe sterility, i.e., defective centromeres [47], implying that altering the N-terminal tail composition has the potential to create haploid inducer. In addition to the simple conjugation of the GFP tag at the N-terminus (GFP Fusion, Fig. 1), replacement of the N-terminal tail with H3.3 N-terminal tails with or without a GFP tag has been shown to induce haploid formation [32, 47, 48] (Tail Swap, Fig. 1). In addition, replacement of the N-terminal tail of *A. thaliana* CENH3 with that of the mustard family species *Lepidium oleraceum* can induce haploids (Tail Swap, Fig. 1) [40]. Attempts by N-terminal tail editing have been performed in diverse species [49], and HI has been successfully achieved in maize [48], tomato and rice [50]. Although the HI rates are relatively low (0.065–0.86% in maize, 0.2–2.3% in tomato, and 0.3–1.0% in rice), these experiments demonstrated the feasibility of HI by engineering the CENH3 N-terminal tail in monocotyledonous crop plants.

### C-terminal histone fold domain editing

A portion of the C-terminal histone fold domain (HFD) that includes loop1 and the α2 helix, known as CATD (Fig. 1), is essential for centromere targeting [44, 51]. The substitution of CATD enabled the incorporation of an H3 chimera into the centromere in *Drosophila* [45]. Mutations in the CENP-A HFD loop1 residues R80 and G81 and CID (*Drosophila* CENH3) D211 lead to reduced CENP-A and CID retention in their respective centromeres [52, 53]. These results imply that modification of the HFD may create a haploid inducer. This strategy was proven to be feasible by recent studies from two labs [37, 38]. Karimi-Ashtiyani et al. created point mutations of residue L130 in *A. thaliana* CENH3 HFD. The single-insertion line, *Atcenh3* L130F induced haploids when crossed with WT plants (HFD Point Mutation, Fig. 1). Meanwhile, Kuppu et al. adopted a systematic strategy to screen potential HFD residues, determining which can result in zygotic missegregation in combination with WT CENH3. They first identified conserved amino acids from the HFDs of *A. thaliana*, *B. rapa*, *Solanum lycopersicum* and *Zea mays* and predicted functionally tolerated amino acid substitutions. After transgenic function assays, five point-mutations, P82S, G83E, A132T, A136T, and A86 V, were shown to produce paternal haploids at the rate of 0.61–12.2%.

Unlike the tailswap-CENH3 haploid inducer, all of these point-mutant plants were viable and fully fertile when self-pollinated, without any obvious phenotypic effects [37, 38]. These fully viable and fertile inducer options will facilitate the application of this technique and statistically improve HI rate, because many more fertile gametes are generated from a single plant than from the N-terminal chimeric version of GFP-tailswap (most male sterile and ~ 60% female fertility) [32].

### CENH3 replacement

Although highly variable in sequence, CENH3 has essential functions that are conserved across a broad evolutionary landscape in eukaryotes [40], as first proven by the finding that yeast Cse4 (CENH3) can functionally replace CENP-A in humans [54]. Studies in *Arabidopsis* have also shown functional conservation across a broad evolutionary distance, as the exogenous CENH3s from a wide range of species can target *A. thaliana* centromeres [40, 51, 55]. Interestingly, given this functional complementation, CENH3 from *B. rapa* or *L. oleraceum* were found to be ‘noncompetitive’ versus WT CENH3, generating haploids, aneuploids and chromosomal rearrangements [40]. This result revealed the possibility of creating a haploid inducer by completely replacing CENH3 with a CENH3 from a related species. Like the HFD point-mutant individuals, the transgenic *Arabidopsis* carrying CENH3 from *B. rapa* or *L. oleraceum* were phenotypically indistinguishable from WT and were also self-fertile, which compensate for its low HI rates (1–11%).

## Mechanism of centromere-mediated haploid induction

A consistent phenomenon in the centromere-mediated HI is that the haploid plants contain only WT chromosomes. Moreover, no haploids were obtained from self-fertilized *CENH3* mutant plants and plants with coexpressed WT and mutant *CENH3* genes. These results indicate that the modified CENH3 has weakened centromere function, which leads to uniparental chromosome loss when competing with WT CENH3 during mitosis [32, 39]. Recently, a centromere-size model provided a plausible explanation for the elimination of chromosomes with defective CENH3 [56]. Briefly, defective CENH3 is expected to have a lower efficiency in recruiting some key kinetochore proteins, leading to the smaller centromere sizes in outcrosses. These smaller centromeres have less accurate metaphase plate alignment, causing inefficient recruitment of centromeric factors and leading to a high level of stochastic chromosome loss. The findings that tailswap- and point-mutant CENH3s lead to reduced centromere loading [37, 47] provide indirect evidence to support the above assumption. Furthermore, when an inducer is self-pollinated, all centromeres are on an equal defective level and thus are competitively retained. Thus, the centromere-size model can also explain the observation that homozygous tailswap-CENH3 and other inducer lines are self-fertile and do not produce haploids. In contrast, small centromeres that can expand to match the average size of other centromeres will be retained in the progeny, producing aneuploid or diploid individuals.

This centromere-size model is derived from the concept that each centromere has a similar size within a species [57, 58]. Zhang and Dawe [57] examined ten species of grass and observed similar immunoassay signal intensities from each centromere that suggested uniform centromere size within a species. Studies through chromatin immunoprecipitation followed by sequencing (ChIP-seq) using an anti-CENH3 antibody confirmed the uniform size of centromeres within a species [59–63]. However, electron microscopy and immunoassay observation analyses revealed that centromere sizes across species are different and correlate with genome size and total centromere volume [57, 64]. Therefore, the similar size of maize and oat centromeres observed in oat-maize addition lines indicates that the alien maize centromeres have adopted a similar size to oat centromeres. This hypothesis was confirmed by the analyses of anti-CENH3 ChIP-seq in eight oat-maize addition lines [59]. Thus, the retention of the maize chromosomes in oat-maize addition lines can be attributed to expansions in centromere size allowing them to match the average size of the other centromeres, whereas failure of the smaller centromeres from one parent to adopt the required size may cause genome elimination in a hybrid.

In fact, during wide hybridizations between a large-genome species (such as oat, barley and wheat) and a small-genome species (such as maize, pearl millet, adlay millet, perennial rye grass or sorghum), chromosomes from the small-genome parent are often eliminated in early embryogenesis [65–67]. Thus, these findings not only support the centromere-size model but also suggest that the centromere-size model can explain the HI caused by wide hybridization.

## Competitive CENH3 loading may influence chromosome retention or loss

**Fig. 2:**
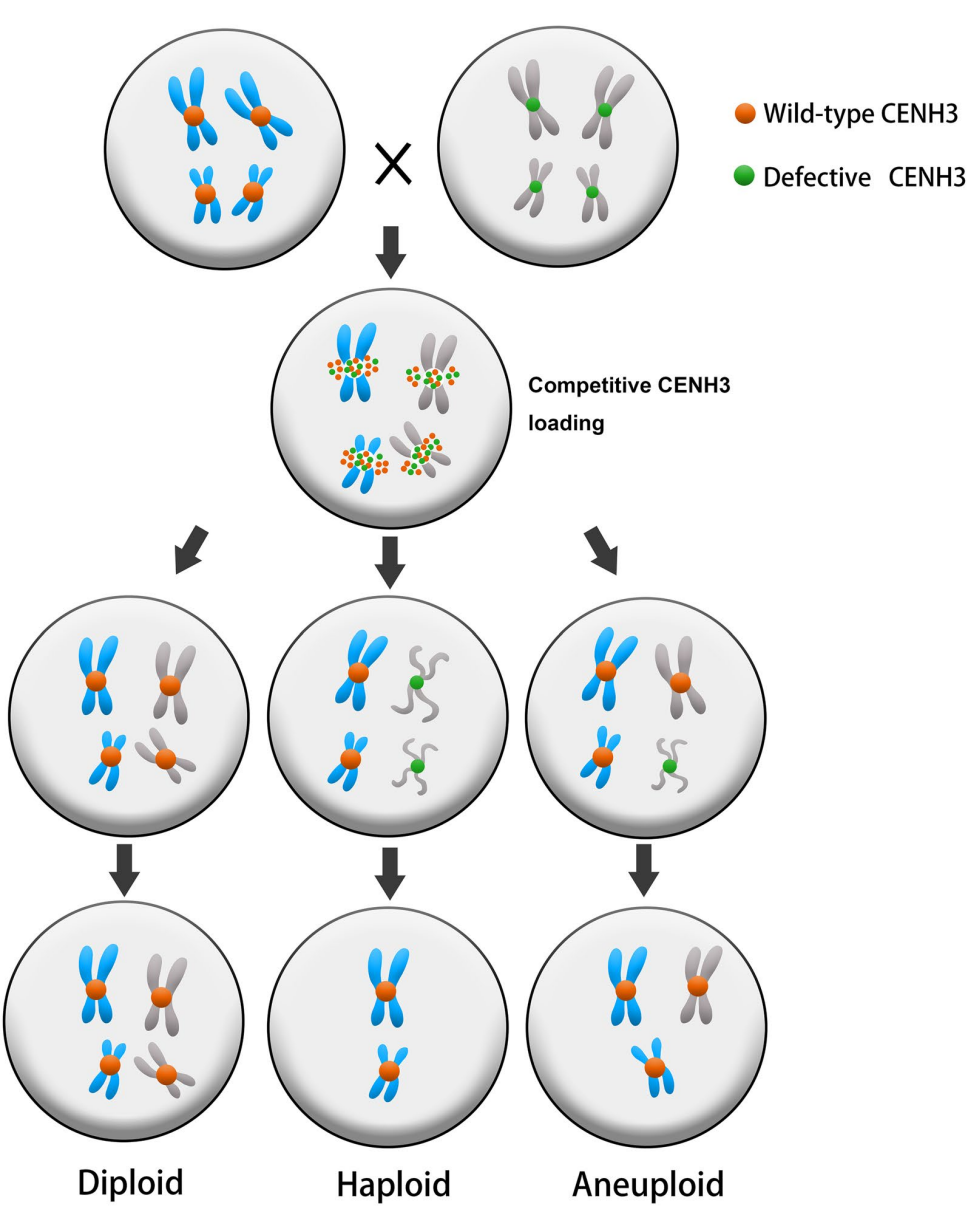
The model of centromere-mediated chromosome elimination. According to the centromere-size model, CENH3-mediated HI involves cross between a wild-type line and a haploid inducer line with smaller/defective centromeres. A competitive loading process between WT and mutant CENH3s occurs during the hybrid formation. WT CENH3 may competitively load to all centromeres, thus generating a uniform centromere size and producing diploid progenies. In contrast, haploid inducer-derived centromeres may load with defective CENH3, generating smaller or defective centromeres, and they would be eliminated, producing haploid or aneuploid offspring

An intriguing finding is that plants with coexpressed WT and mutant *CENH3* genes (*GFP*-*tailswap*, *GFP*-*CENH3* and *Hvßcenh3* L92F) do not act as haploid inducers, because no haploid or hypoploid plants are obtained [32, 37]. According to the centromere-size model, this lack of HI suggests that all the centromeres should have a uniform size in those hybrid plants. A reasonable explanation is that WT CENH3 may competitively load to all centromeres, thus generating a uniform centromere size and producing diploid progenies. In contrast, if the WT and mutant CENH3s were constrained to load into the corresponding WT- and mutant-derived centromeres, it was expected to see haploid progeny, because the centromeres loaded with mutant CENH3 would be smaller, and they would be eliminated, producing haploid or aneuploid offspring. Therefore, the lack of HI in plants with coexpressed WT and mutant *CENH3* genes suggests the possibility that a competitive loading process between WT and mutant CENH3s occurs (Fig. 2). In oat-maize addition lines, the additional maize centromeres are consistently incorporated by oat CENH3 [68] and adopt a similar size to the oat centromeres [59]. In addition, HTR12 (*A. thaliana* CENH3) can be incorporated into the centromeres of *A. arenosa* in an allopolyploid of *A. thaliana* and *A. arenosa* [69]. All these findings support our hypothesis.

The WT and mutant-derived centromeres should retain their corresponding WT and modified CENH3s in early zygotic mitoses, because CENH3 can be sustained through mitosis after the initial steps of centromere assembly [70, 71] (Fig. 2). These defective mutant CENH3s continuously target the inducer-derived centromeres and produce smaller centromeres due to their inefficient centromere loading procedure. These smaller centromeres are noncompetitive and are later eliminated when encountering WT centromeres. However, mutant CENH3 loading must occur in a short time frame early in the zygotic mitoses. Alternatively, the WT CENH3 would competitively load into some or all of the mutant-derived centromeres, producing normal large-sized centromeres and generating normal diploid or aneuploid plants. Clearly, our revised model is consistent with and based on the centromere-size model, but it also presents a plausible explanation for both HI incapability despite co-ocurrence of WT and mutant CENH3s [32, 37] and CENH3 loading in the centromeres of alien species in stable hybrids [68, 69]. This hypothesis is testable through further centromere loading assays involving WT and mutant CENH3s during the early stage of zygotic development.

## Phospholipase-triggered haploid induction in maize

In maize, the line Stock6 has been widely used to generate haploids for inbred line production. The original Stock6 inducer line was created by Coe in 1959 with HI rates of 2.52% and 1–2% when Stock6 was selfed or outcrossed as a male, respectively [72]. Selective breeding has improved the HI capability of Stock6 derivatives to 7–15% [73, 74]. Several quantitative trait loci (QTLs) associated with HI have been identified, indicating that the HI in Stock6 is controlled by multiple genes [75–79]. Among those candidate QTLs, *qhir1*, which is located in bin 1.04, was shown to explain 66% of the genetic variance and has attracted the most attention.

Researchers from Syngenta conducted the first trial to isolate the HI gene from the *qhir1* region [80]. After fine mapping, they narrowed the QTL to a 0.57 Mb region containing 7 genes. Sequence comparisons revealed that the gene *GRMZM2G471240* (*MATRILINEAL*) has a 4-bp insertion (CGAG) in the induction lines compared to the B73 genome. This 4-bp insertion in the fourth exon leads to a frame shift, causing 20 altered amino acids and a premature transcription termination that truncates the protein by 29 amino acids. After functional verification (gene knockdown and knockout), they verified that *MTL* was responsible for HI. Two other teams from China and France also achieved similar results after sequence assay and gene-editing validation (referred *GRMZM2G471240* as *ZmPLA1* and *NLD*, respectively) [81, 82]. Notably, large-scale sequence comparisons revealed that the 4-bp insertion was a distinct feature restricted to only the inducer lines, confirming its critical role in HI [81]. In addition, the absence of such a 4-bp insertion from the ancestral variety, teosinte, suggested that this mutation occurred after maize domestication [81]. Another interesting finding is that some gene-editing and knockdown events increased HI rates, suggesting that it is possible to create a high-HI-rate inducer by modifying *MTL*.

*MTL* encodes a patatin-like phospholipase and is expressed specifically in maize pollen [80–82]. Phospholipase alterations are associated with delayed pollen germination and pollen tube growth [83], which explain the pleiotropic phenotypes accompanying with maize HI capability [73, 80]. However, the mechanisms of HI that contribute to single fertilization [84] or postzygotic genome elimination [85, 86] remain unclear. An RNA-seq assay showed that genes associated with pollen cell endomembrane and lipid composition or Ca^2+^-involved signaling pathways have potential effects on *mtl* HI [80]. A subcellular localization assay revealed that the MTL protein targets the sperm cell plasma membrane in both maize and *Arabidopsis*. In contrast, the truncated protein from the inducer line PK6 was absent from the plasma membrane [80]. Further in silico analysis of the MTL protein demonstrated that the absence of a lipid anchor site from the truncated protein C-terminal may contribute to its mislocalization. Collectively, these results indicate that membrane integrity or mechanisms involving signaling precursor triggered by defective MTL might be responsible for HI capacity [82].

Recently, Li et al. [87] discovered that approximately 10% of kernels were aborted when pollinated with the inducer line, which has almost no detectable kernel abortion as a female parent. The proportion of viable inducer pollen is lower than that of inbred lines, suggesting that defective pollen development may be attributed to HI. After single-nucleus sequencing of mature pollen, they demonstrated that chromosome fragmentation that begins around pollen mitosis may be the cue causing pollen abortion and HI. Given that the haploid is an abortive kernel with a haploid embryo, only those that initiate fragmentation after the 2nd mitosis can generate haploid nonaborted kernels [87]. However, detailed embryo development studies still need to be performed, because the possibility of single fertilization (fragmented sperm not fusing with the egg) cannot be excluded [87]. However, the results of Li et al. shed light on either the mechanism or the development of new HI superinducer. As HI is potentially caused by pollen sperm fragmentation after the 2nd mitosis, increasing the frequency of chromosome fragmentation initiated after the 2nd mitosis could potentially improve the HI rate. Alternatively, decreasing early chromosome fragmentation, before the 2nd mitosis, would be an efficient strategy to improve kernel viability.

## Conclusions and perspectives

The CENH3 function is highly conserved across eukaryotes. Moreover, the centromere is composed of multiple proteins, which means that other key centromere proteins, in addition to CENH3, are also expected to be potential targets for HI inducer development. Thus, CENH3-based HI has tremendous potential for successful application in any plant theoretically. To date, in addition to the model plant *Arabidopsis*, haploids have been successfully induced in maize [48] using the tailswap strategy. However, it is perplexing that the CENH3 tailswap plants in maize did not induce haploids as frequently as in *Arabidopsis* [32]. Due to the enigmatic structure of centromeres [88], functional divergence of CENH3s in other plants compared with the dicot model plant *Arabidopsis* is possible. Supporting the hypothesis, the *Arabidopsis* tailswap inducer shows severe sterility (mostly male sterile and 60–70% female fertile) [32] while that was not observed in maize [48]. It therefore implied that the HI ability might be correlated with the plant fertility. It is plausible that haploid inducer with more severe defective CENH3 will likely cause both more severe sterility and higher HI rate because the mutated CENH3 can not complement the full function of CENH3 and is less competitive when encountering with wild CENH3. In fact, the plant with the highest HI rate was also the plant with the strongest knockdown of *CENH3* in maize [48] supporting the above hypothesis. Therefore, it will be interesting to explore the correlation of inducer sterility and HI rate. Moreover, the usage of WT parent is another factor that should be considered since the HI rates can be nearly two-fold change when different WT lines were crossed with the same inducer in *Arabidopsis* [32]. Theoretically, given that the HI rated are still low using the tailswap strategy in crops, the methods of HFD modification, CENH3 replacement and genetic editing other kinetochore components provide alternative choices for the development of inducers with high HI rates. Importantly, point mutations can be achieved by nontransgenic chemical mutagenesis, and then, the results can be directly used in crop breeding.

The centromere-size model provides a basis to explain HI resulting from both wide hybridization and CENH3-mediated systems. Our CENH3 competitive loading model helps to explain the observation that inducer lines lose HI capability when both WT and mutant CENH3s are present. This hypothesis is expected to be easily tested by further WT and modified CENH3 targeting assays. Based on the centromere-size model, crosses between inducer and larger-centromere lines should produce haploids with high efficiency. Thus, this model will allow us to predict HI capacity before crossing experiments, which will greatly improve the application of HI to crop breeding.

**Fig. 3:**
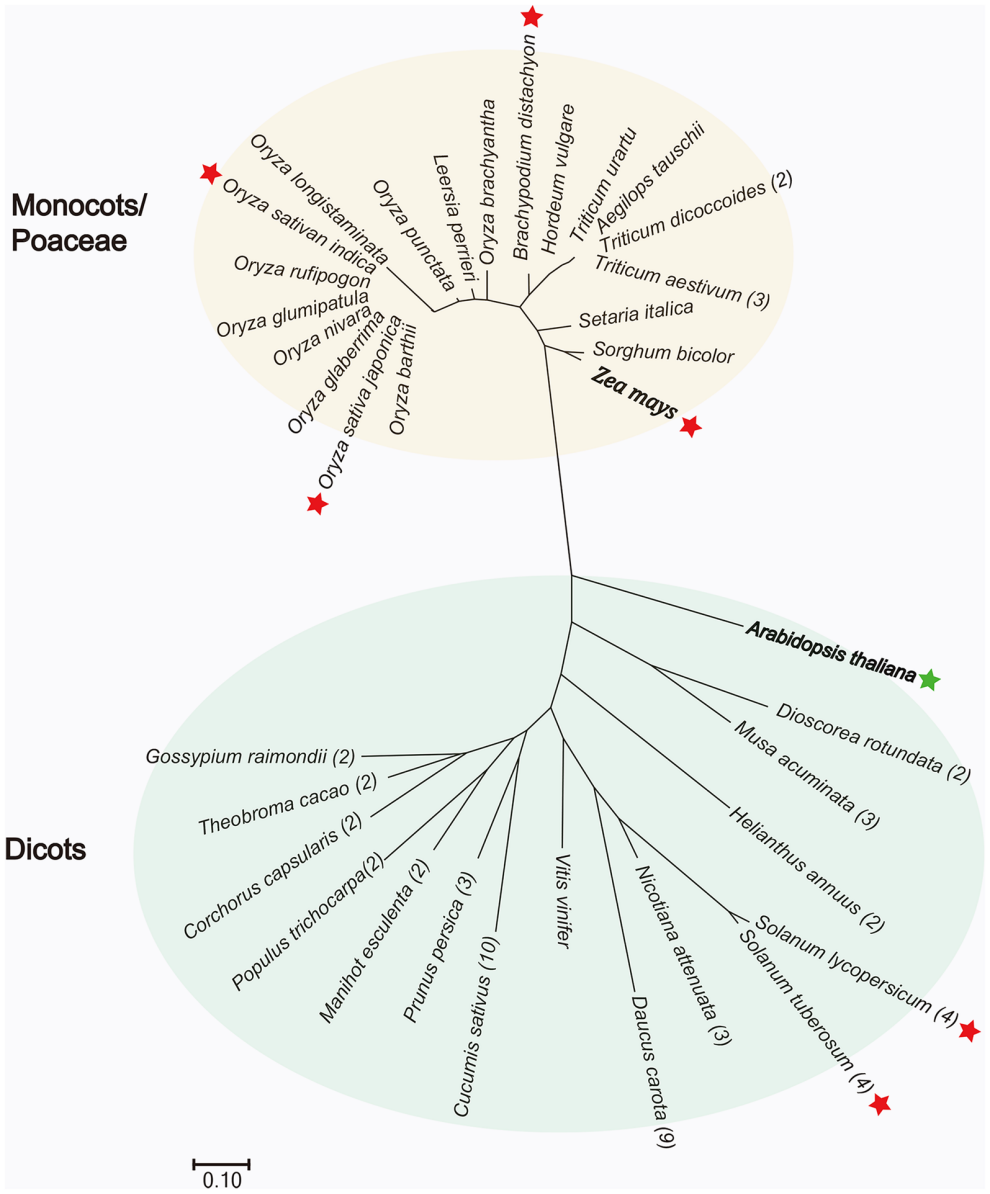
Phylogenetic analysis of *MTL* gene orthologs in plant. The available orthologs of *MTL* genes were collected and analyzed using the Neighbor-Joining method. The orthologs which showed expression pattern in reproductive organ, were highlighted by asterisk. Copy numbers of *MTL* orthologs were indicated in the brackets

The HI capacity of maize Stock6 is caused by at least seven potential genes (or QTLs) [75, 79], indicating that a potentially enhanced HI inducer could be created by combining multiple HI-related genes. Thus, it is worthwhile to devote effort to the identification of other major HI-associated QTLs/genes, such as *qhir8*, which was found to explain 20% of the genotypic variance [89]. In addition, alteration of the *MTL* gene renders it possible to extend this tool to other species, at least in *Poaceae* [80, 90], because of the high-level conservation of *MTL* orthologs (Fig. 3). Its successful application in rice is very encouraging [91]. However, the presence of numerous co-orthologs [82] and non-pollen-specific expression in dicots [92] (Fig. 3, Additional file 1: Table S1) raises some uncertainty about its utility in those or other plants. Thus, a careful function(s) assessment of orthologs will be required before experimental trials in other species.

Notably, the CENH3-mediated inducer can induce haploids as both female and male. This feature provides it with wide versatility, permitting diploid production from individuals with the desired genomic backgrounds. In addition, crosses using the inducer as a female will transfer the nuclear genome of the male parent to a heterologous cytoplasm, which will potentially facilitate the generation of cytoplasmic male sterile lines [32]. In contrast, the classical *MTL*-mediated Stock6 and its derivatives induce exotic-parent haploids only as male (although haploids can be induced on self-pollination), which will generate only haploids with homologous cytoplasmic and nuclear genomes. Nevertheless, the evidence from maize demonstrates that this issue can be solved by the CENH3-mediated HI system [48], which has potential utility unlimited by plant species.

## Additional file

**Additional file 1: Table S1.**The information of MTL orthologs.

## Abbreviations

CENH3: centromere histone H3
HI: haploid induction
*MTL*: *MATRILINEAL*
WT: wild-type
GFP: green fluorescent protein
ChIP-seq: chromatin immunoprecipitation followed by sequencing
HFD: histone fold domain
QTLs: quantitative trait loci
