# Supplementary material for: Centromere histone H3- and phospholipase-mediated haploid induction in plants
Source: Plant Methods. 2019 Apr 26;15:42. doi: 10.1186/s13007-019-0429-5 (PMC6485145; doi:10.1186/s13007-019-0429-5)
Supplement: Supplementary file 1 — Additional file 1: Table S1.The information of MTL orthologs. [file 13007_2019_429_MOESM1_ESM.docx]

**Table S1 The information of *MTL* orthologs.**

| Species | Genbank ID/Gene ID | Reference |
| --- | --- | --- |
| *Sorghum bicolor* | EER94748 |  |
| *Aegilops tauschii* | EMT15946 |  |
| *Arabidopsis thaliana* | At2g26560 | Camera et al. 2005 |
| *Brachypodium distachyon* | KQK21408 |  |
| *Corchorus capsularis* | OMO56728 |  |
| *Cucumis sativus* | KGN62054 |  |
| *Daucus carota* | KZM88636 |  |
| *Dioscorea rotundata* | Dr12875 |  |
| *Gossypium raimondii* | KJB71085 |  |
| *Helianthus annuus* | OTF96063 |  |
| *Hordeum vulgare* | HORVU4Hr1G074979 |  |
| *Leersia perrieri* | LPERR03G17310 |  |
| *Manihot esculenta* | OAY35344 |  |
| *Musa acuminata* | Achr7P11810_001 |  |
| *Nicotiana attenuata* | OIT22532 |  |
| *Oryza barthii* | OBART03G20280 |  |
| *Oryza brachyantha* | OB03G29460 |  |
| *Oryza glaberrima* | ORGLA03G0189400 |  |
| *Oryza glumipatula* | OGLUM03G20950 |  |
| *Oryza longistaminata* | KN538811.1_FGP010 |  |
| *Oryza nivara* | ONIVA03G21950 |  |
| *Oryza punctata* | OPUNC03G19040.1 |  |
| *Oryza rufipogon* | ORUFI03G20930.1 |  |
| *Oryza stiva indica* | BGIOSGA012798-PA | Li et al. 2018 |
| *Oryza stiva japonica* | LOC_Os03g27610 | Singh et al. 2012 |
| *Populus trichocarpa* | PNT26993 |  |
| *Prunus persica* | ONH96560 |  |
| *Setaria italica* | KQK905590 |  |
| *Solanum lycopersicum* | Solyc02g065090.2.1 | Matas et al.(2011) |
| *Solanum tuberosum* | Kalax.0244s0015.1 | Vancanneyt et al (1989) |
| *Theobroma cacao* | EOX91345 |  |
| *Triticum aestivum* | TraesCS4A02G018100.1 |  |
| *Triticum urartu* | TRIUR3_10816-P1 |  |
| *Triticum dicoccoides* | TRIDC4AG002450.1 |  |
| *Vitis vinifera* | VIT_00s0567g00060.t01 |  |
| *Zea mays* | GRMZM2G471240 | Kelliher et al.2017 |

**Reference:**

Camera S L , Geoffroy P , Samaha H , et al. A pathogen-inducible patatin-like lipid acyl hydrolase facilitates fungal and bacterial host colonization in Arabidopsis[J]. Plant Journal, 2005, 44(5):810-825.

Li Y , Ya Z , Chunxia L , et al. OsMATL mutation induces haploid seed formation in indica rice[J]. Nature Plants, 2018.

Singh A , Baranwal V , Shankar A , et al. Rice Phospholipase A Superfamily: Organization, Phylogenetic and Expression Analysis during Abiotic Stresses and Development[J]. PLoS ONE, 2012, 7(2):e30947.

Matas A J , Yeats T H , Buda G J , et al. Tissue- and Cell-Type Specific Transcriptome Profiling of Expanding Tomato Fruit Provides Insights into Metabolic and Regulatory Specialization and Cuticle Formation. The Plant Cell, 2011, 23(11):3893-3910.

Vancanneyt, G. Expression of a Patatin-like Protein in the Anthers of Potato and Sweet Pepper Flowers[J]. THE PLANT CELL ONLINE, 1989, 1(5):533-540.

Kelliher T , Starr D , Richbourg L , et al. MATRILINEAL, a sperm-specific phospholipase, triggers maize haploid induction[J]. Nature, 2017, 542(7639):105-109.
